# Design of topology optimized compliant legs for bio-inspired quadruped robots

**DOI:** 10.1038/s41598-023-32106-5

**Published:** 2023-03-25

**Authors:** Yilun Sun, Chujun Zong, Felix Pancheri, Tong Chen, Tim C. Lueth

**Affiliations:** 1 Institute of Micro Technology and Medical Device Technology, Technical University of Munich, 85748 Garching, Germany; 2 Institute of Energy Efficient and Sustainable Design and Building, Technical University of Munich, 80333 Munich, Germany; 3 Department of Precision Engineering, School of Engineering, The University of Tokyo, Tokyo, 113-8656 Japan

**Keywords:** Mechanical engineering, Electrical and electronic engineering

## Abstract

Robotic legs are an important component of the quadruped robot for achieving different motion gaits. Although the conventional rigid-link-based legs can generally perform robust motions, they still have the issues with poor sealing when operating in complex and liquid terrains. To cope with this problem, fully compliant legs with monolithic structure have been introduced in recent years to improve the system compactness and structural compliance of quadruped robots. In this article, we present a topology-optimization-based method to achieve efficient design of compliant robotic legs. In order to balance the structural stiffness and bending flexibility of the realized leg, a multi-objective optimization algorithm is utilized. A series of design cases are presented to illustrate the design principle and analytical procedure of the proposed method. In addition, experimental evaluation is also performed, and the results have demonstrated that, a quadruped robot with the optimized legs can successfully achieve stable and continuous straight-line walking motions.

## Introduction

The quadruped robot is a kind of mobile robot whose locomotion is inspired by four-legged animals in nature, such as dogs, horses, and cheetahs. Compared with wheeled robots, quadruped robots are more adaptable to different terrains^1^, which makes them suitable for performing complex exploration tasks in unstructured and dangerous environments. Based on this advantage, a variety of quadruped robots have been developed in recent decades to achieve different locomotion gaits and applications. For example, Spröwitz *et al.* have designed a compliant quadruped robot Cheetah-Cub^2^ to achieve fast and dynamic trotting gaits with different speeds. Another quadruped robot, the MIT Cheetah 2^3^, has realized stable bounding gaits by directly controlling the ground reaction forces. To cope with the locomotion tasks in multiple environments, several amphibious quadruped robots, such as the robotic turtle ART^4^ and the robotic swimming dog^5^, were also developed, which can operate both on land and in water. Other kinds of multi-functional quadruped robots, for instance the ANYmal^6^, are capable of autonomously performing different complex tasks, such as industrial inspections and rescue missions in natural disasters.

As a key component of quadruped robots, the mechanical legs play an important role in achieving different locomotion gaits. In order to analyze their mechanical performance, we can classify the legs of conventional quadruped robots into three categories^7^: prismatic, articulated, and redundant articulated legs. Basically, the motion of a prismatic leg is realized by using a rotating joint and a linear prismatic joint, while the articulated leg utilizes two rotating joints to achieve the two degree-of-freedom (DOF) motion. The redundant articulated leg, on the other hand, has introduced another rotating joint and supporting springs into the 2-DOF articulated leg to improve its motion stability and energy use efficiency. In the current state of the art, most of these three types of legs^2,3,6,8,9^ have employed rigid-link-based joints for motion transmission. Although the rigid-link mechanisms are generally stable and robust, they still suffer from poor sealing when interacting with complex environments, such as flooded terrains and deserts. To cope with this problem, compliant (or soft) legs with bio-inspired monolithic structures have been recently introduced to the design of quadruped robots. For example, Tolley *et al.*^10^ have developed a quadruped soft robot using silicone elastomer material and pneumatic actuation. With the appropriate combinations of the 1-DOF bending motions of each leg, the created robot can perform stable crawling gaits in various environments. Other researchers also developed soft robotic legs^5,11^ with pre-charged pneumatic structures and tendon-driven mechanisms to achieve complex leg motions. In addition to silicone-based molding, three-dimensional (3D) printing technology were also used in some studies^12–14^ to fabricate fully compliant legs for bio-inspired quadruped robots.

Due to the monolithic structure of the compliant robotic legs, it is difficult to use the traditional rigid-link theory to achieve their mechanism design. To cope with this problem, there are mainly two methods from the literature that are frequently used for designing compliant mechanisms, which are the pseudo-rigid-body-model (PRBM) method^15^ and the topology optimization method^16^. Using the PRBM method, the flexible joints in the compliant leg are simplified as torsional springs between rigid links. With this assumption, the rigid-link theory can be modified for the mechanism synthesis of compliant legs. However, the PRBM-based synthesis method does not fully explore the design freedom of compliant mechanisms because the design results largely depends on the predefined mechanism models. From this point of view, the bio-inspired topology optimization method provides a more general solution, as it can automatically find the optimal mechanism topology from the design domain that fulfills the design requirements. Therefore, in this article, we propose a topology-optimization-based framework to achieve efficient design of the compliant legs of quadruped robots. This work has the following contributions:

1. Development of a multi-objective topology optimization method that takes into account both the standing and walking positions of the compliant leg.
2. Analysis of the effect of different design parameters on the performance of the optimized legs.
3. 3D printing of a quadruped robot with the optimized legs and experimental evaluation of its locomotion performance.

The remainder of this article is organized as follows. “Methodology” section provides a detailed description of the multi-objective topology optimization method for the leg design. The shape of the optimized legs and the parameter analysis are presented in “Optimization results” section. “Experiment” section shows the experimental results of the 3D-printed quadruped robot with optimized legs. In “Discussion” section, the performance of the proposed method is discussed, and future work is also outlined.

## Methodology

### Overview of the topology optimization method

**Figure 1:**
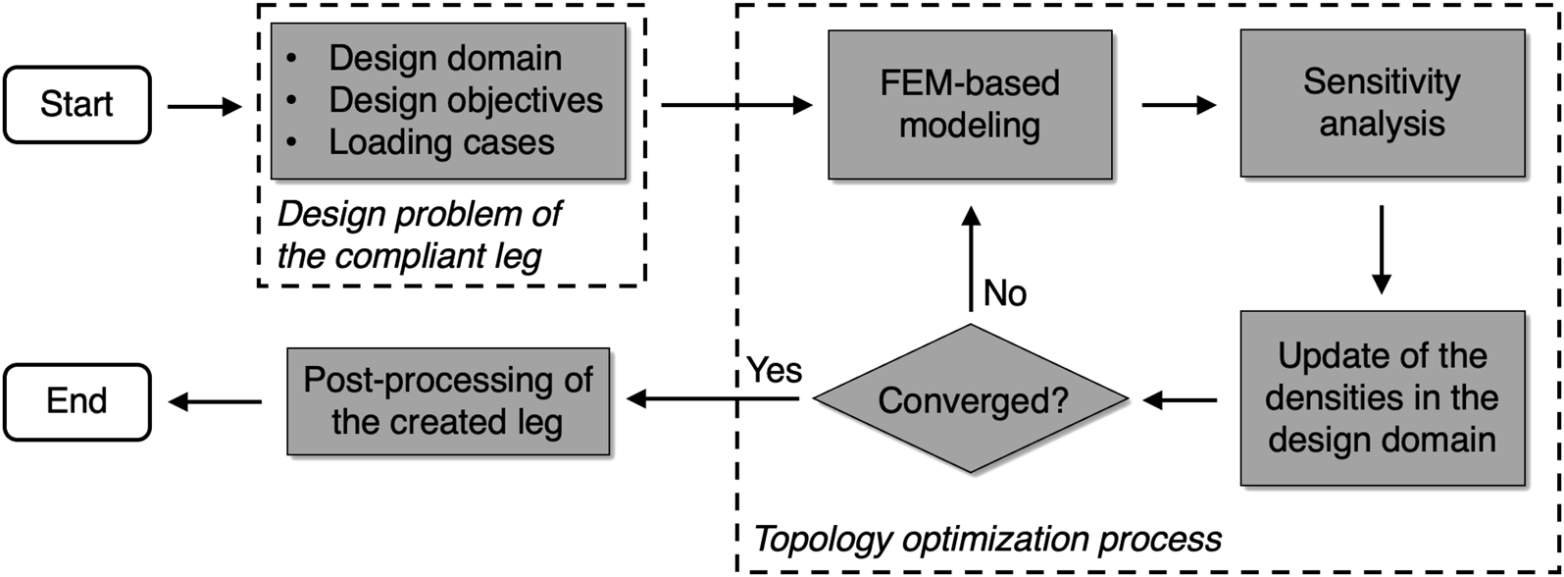
Workflow of the proposed topology-optimization-based design method.

Figure 1 shows the generalized workflow of the proposed design method. The first step is to formulate the design problem for the compliant leg, which includes the geometrical design domain, the multiple design objectives and the related loading cases. In the second step (the iterative topology optimization process in Fig. 1), the finite element method (FEM) is used in each iteration to mesh the 3D design domain and calculate the deformations of the leg. With the FEM-calculated results, the sensitivity analysis is performed and an update scheme is used to iteratively modify the material densities in the design domain until the optimization process converges. In the last step, post-processing techniques are used to generate a 3D-printable leg model from the optimized density data. The entire method is implemented in a unified design framework in MATLAB^17,18^, which is under development at our institute.

### Formulation of the design problem

In this work, we have utilized a 3D cuboid as the design domain for the compliant leg. By using the FEM method, the design domain can be meshed into $N_e$ hexahedral elements. The relationship between the applied load and the induced deformation of the leg can be expressed as:

1 $$\begin{aligned} \mathbf {KU = F} \quad \text{ with } \quad {\textbf{K}}=\sum \limits _{e=1}^{N_e}x_e^p \cdot \mathbf {K_e} \end{aligned}$$

where ${\textbf{F}}$ and ${\textbf{U}}$ are the load vector and the displacement vector, respectively. ${\textbf{K}}$ is the global stiffness matrix obtained by assembling the elemental stiffness matrix $\mathbf {K_e}$ of all elements. Here, $x_e \in (0,1]$ is the normalized density of each element, which serves as the design variable of the optimization process. With the penalty parameter *p*, $x_e$ can be used to modify the material stiffness in the design domain^19^. In this work, we aim to achieve a collection of optimized densities $x_e$ with a solid-void (1 or 0) distribution.

**Figure 2:**
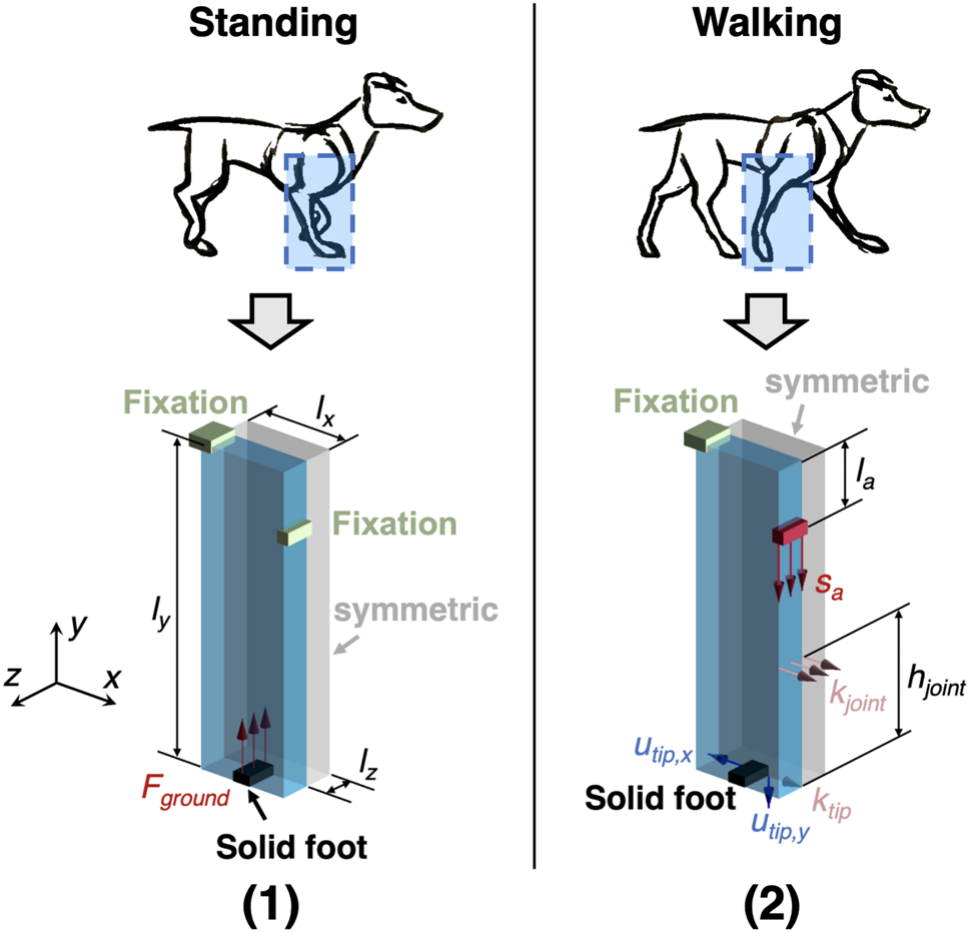
Two loading cases of the compliant leg for the proposed multi-objective topology optimization method: (1) The standing position of the leg, (2) The walking (bending) position of the leg.

In order to achieve a robotic leg with high flexibility and also sufficient structural stiffness, we have included two design objectives in the design problem: (1) The leg should have high structural stiffness (low structural compliance) in its standing position; (2) The leg should perform a 1-DOF bending motion when an actuation force is applied on it. Accordingly, the design problem can be mathematically formulated as:

2 $$\begin{aligned} \left. \begin{aligned} \min _{{\textbf{x}}}&: f_1({\textbf{x}}) = \mathbf {F_1^{T}U_1} \\ \min _{{\textbf{x}}}&: f_2({\textbf{x}}) = -\mathbf {L^{T}U_2} \\ \text {s.t.}&: \mathbf {K_1 U_1=F_1} \text{, } \mathbf {K_2 U_2=F_2} \\&: \frac{\sum \nolimits _{e=1}^{N_e} v_e x_{e}}{V_0} \le c \end{aligned} \right\} \end{aligned}$$

where the objective functions $f_1({\textbf{x}})$ and $f_2({\textbf{x}})$ represent (1) the overall structural compliance $\mathbf {F_1^{T}U_1}$ of the leg in its standing position and (2) the foot motion $\mathbf {L^{T}U_2}$ of the leg in its walking position, respectively. ${\textbf{x}}$ is a vector containing all $x_e$. Herein, we aim to achieve a Pareto optimality of a minimum $\mathbf {F_1^{T}U_1}$ and a maximum $\mathbf {L^{T}U_2}$ (minimum of $-\mathbf {L^{T}U_2}$). In addition, a volume constraint *c* is also introduced to control the final volume of the optimized leg, where $V_0$ and $v_e$ are the volume of the initial design domain and a single element, respectively.

The boundary conditions and loading cases for the two design objectives are illustrated in Fig. 2. Here, the side surface of the design domain (blue) in both cases is set as a symmetric surface because we expect to realize a symmetric leg. A solid foot ($x_e = 1$) is also introduced to ensure that the realized leg has sufficient contact surface with the ground. In the first case [the standing position in Fig. 2(1)], two fixation bases (green) are used to fix the leg to the main body of the quadruped robot, while $F_{ground}$ is used to represent the supporting force from the ground. By incorporating $F_{ground}$ into the load vector $\mathbf {F_1}$, the displacement vector $\mathbf {U_1}$ can be obtained from $\mathbf {K_1} \mathbf {U_1}=\mathbf {F_1}$. In the second case [the walking position in Fig. 2(2)], the utilized design domain is the same as in the first case, while a motor-generated input displacement $s_a$ is introduced in the load vector $\mathbf {F_2}$ to replace $F_{ground}$, with the aim to bend the leg. Here, the length $l_a$ is reserved for the construction of a rigid rod to connect the motor and the leg. The induced x-axis and y-axis displacement of the foot tip, $u_{tip,x}$ and $u_{tip,y}$, are then chosen to describe the foot motion of the leg, since we expect that the realized leg can step down and backwards at the same time to move the robot forward. In this way, the foot motion term in $f_2({\textbf{x}})$ can also be written as:

3 $$\begin{aligned} \mathbf {L^{T}U_2} = u_{tip,x} + u_{tip,y} \end{aligned}$$

where ${\textbf{L}}$, also mentioned in (2), is a sparse vector used to select $u_{tip,x}$ and $u_{tip,y}$ from the global displacement vector $\mathbf {U_2}$. To improve the stress distribution of the bent leg, an additional spring element $k_{joint}$ (in addition to the default tip spring $k_{tip}$) is also integrated into the second loading case, which mimics the structural compliance in the animal bending joints^20,21^. $h_{joint}$ represents the y-axis distance between $k_{joint}$ and the solid foot. As a result, the stiffness matrix $\mathbf {K_2}$ of the leg in the second case can be obtained by modifying $\mathbf {K_1}$:

4 $$\begin{aligned} \mathbf {K_2} = \mathbf {K_1} + \mathbf {K_s} (k_{joint},k_{tip}) \text{, } \mathbf {K_1}=\sum \limits _{e=1}^{N_e}x_e^p \cdot \mathbf {K_e} \end{aligned}$$

where $\mathbf {K_s}$ is the spring stiffness matrix. Then, $\mathbf {U_2}$ can be calculated from $\mathbf {K_2} \mathbf {U_2}=\mathbf {F_2}$.

### Density update and post-processing

To solve the multi-objective design problem in (2), sensitivity analysis is carried out in each iteration for the density update. The sensitivities of $f_1$ and $f_2$ with respect to $x_e$ can be expressed as:

5 $$\begin{aligned} \frac{\partial f_1}{\partial x_e} = -\mathbf {U_1^T}\frac{\partial \mathbf {K_1}}{\partial x_e}\mathbf {U_1} \end{aligned}$$

6 $$\begin{aligned} \frac{\partial f_2}{\partial x_e} = \mathbf {(K_2^{-1} \cdot L)^T}\frac{\partial \mathbf {K_2}}{\partial x_e}\mathbf {U_2} \end{aligned}$$

Based on the results of (5) and (6), we have employed the standard optimality criterion (OC) method^20,22–24^ to update the elemental density $x_e$:

7 $$\begin{aligned} x_e^{new} = x_e \cdot \left[ w_1 \cdot \left( \frac{-\frac{\partial f_1}{\partial x_e}}{\lambda _1 v_e}\right) ^{\eta _1} + w_2 \cdot \left( \frac{-\frac{\partial f_2}{\partial x_e}}{\lambda _2 v_e}\right) ^{\eta _2} \right] \quad \text{ with } \quad w_1 + w_2 = 1 \end{aligned}$$

where $w_1$ and $w_2$ are two prescribed weighting factors for the first and second design objective, respectively. Here, the initial value of $x_e$ is set to *c* and $x_e^{new}$ is the updated density. The Lagrangian multiplier $\lambda _1$ and $\lambda _2$ can be calculated by using a bisection algorithm^22^, while two damping factors $\eta _1 = 0.5$ and $\eta _2 = 0.3$ are introduced for maintaining numerical stability. In order to prevent the checkerboard problem in the optimization process, a sensitivity-based filter^25^ is also used, whose filter radius is correlated to the mesh size. The optimization process terminates when both $f_1$ and $f_2$ converge within a tolerance of $10^{-4}$.

In post-processing, we utilize the iso-surface method^26^ to extract a smooth and 3D-printable surface model from the optimized density vector ${\textbf{x}}$. After that, the selective laser sintering (SLS) technology is used to fabricate the optimized compliant leg.

## Optimization results

In this section, we first carry out a design case to demonstrate the application of the proposed optimization method to the compliant leg design. Then, different values of the volume constraint *c* and the spring position $h_{joint}$ are utilized in the design process to investigate their effect on the mechanical performance of the optimized compliant legs.

### Application to compliant leg design

For the purpose of method verification, a small-scale design domain with the size of $15\, \text {mm} \times 50\, \text {mm} \times 5\, \text {mm}$ was used in this work. To achieve the optimal combination of structural stiffness and bending flexibility, a weighting factor set of [0.3, 0.7] was assigned to the optimization problem, whose selection criterion will be illustrated in the second part of the “Optimization results” section. For the FEM-based modeling, the elastic modulus $E_0$ was set to 1700 MPa as the nylon material (PA2200, EOS GmbH, Germany) was used for the leg prototyping. The spring constant of $k_{joint}$ and $k_{tip}$ were both chosen as 0.5 N/mm according to our preliminary work^20^. In this design case, the spring position $h_{joint}$ and the volume constraint *c* were set to 20 mm and 0.12, while the effect of these two parameters will be further analyzed in the last two parts of the “Optimization results” section. The other parameters used in this design case are also listed in Table 1.

**Table 1:** Parameters used in the optimization process in “Application to compliant leg design” section.

Parameter	Symbol	Value
Size of the design domain	[$l_x$, $l_y$, $l_z$]	[15 mm, 50 mm, 5 mm]
Number of elements in the FE-mesh	$N_e$	30,000
Supporting force in loading case (1)	$F_{ground}$	5 N
Input displacement in loading case (2)	$s_{a}$	5 mm
Position for applying $F_{a}$	$l_{a}$	10mm
Elastic modulus of the material	$E_0$	1700 MPa
Spring constants	[$k_{joint}$, $k_{tip}$]	[0.5 N/mm, 0.5 N/mm]
Weighting factors for the two optimization objectives	[$w_1$, $w_2$]	[0.3, 0.7]

**Figure 3:**
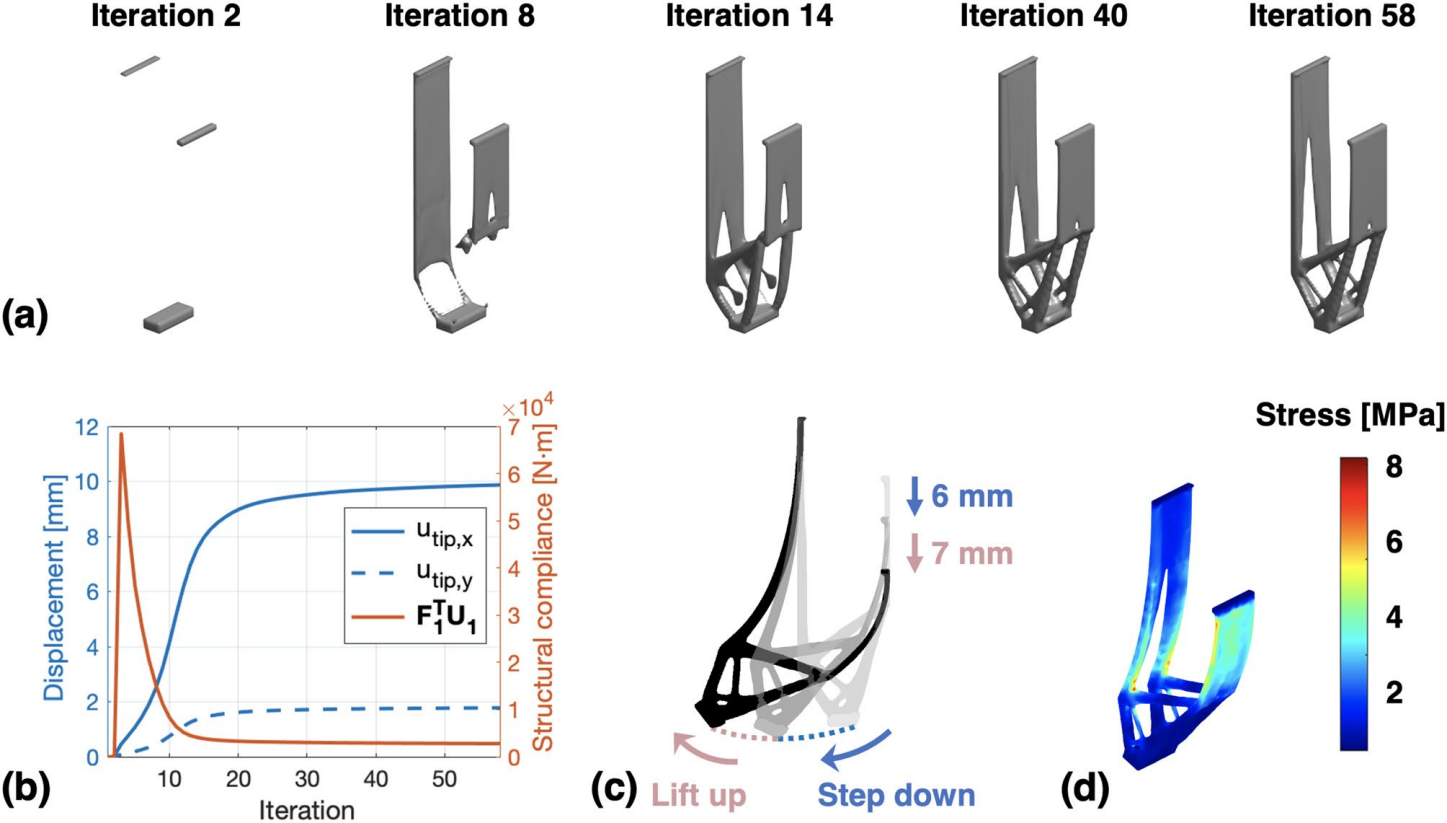
The optimization process and the realized compliant leg in “Application to compliant leg design” section: (**a**) Evolutionary process of the compliant leg shape, (**b**) Trend of the multiple design objectives during the optimization process, (**c**) FEM-simulated bending motion of the optimized leg, (**d**) FEM-simulated stress distribution in the bent leg.

The optimization process and the realized leg shape are presented in Fig. 3. It can be seen that, during the evolutionary process [Fig. 3a], a combination of two flexible beams and a rigid foot structure has emerged in the generated leg geometry. This phenomenon can be interpreted as a balancing strategy of the proposed optimization algorithm for achieving both the bending flexibility and standing stability of the leg. As a result, the Pareto optimality of a minimum structural compliance ($\mathbf {F_1^{T}U_1}$) and a maximum bending displacement ($u_{tip,x} + u_{tip,y}$) is successfully achieved as the optimization process converges [see Fig. 3b]. On the other hand, after performing large-displacement FEM-simulation with $s_a = 13\, \text {mm}$ in Fig. 3c, we can see that the bending motion of the realized compliant leg can be divided into two stages. In the first stage ($0\, \text {mm}<s_a < 6\, \text {mm}$), the foot tip steps down since both $u_{tip,x}$ and $u_{tip,y}$ increase with $s_a$. After that, the foot tip starts to lift up to its original height level as $u_{tip,y}$ decreases with $s_a$ [the second stage ($6\, \text {mm}<s_a < 13\, \text {mm}$)]. This two-stage motion behavior of the realized compliant leg is similar to that of many quadrupeds, which is useful in this work for switching walking legs during the robotic walking motions. Herein, we define the total displacement of $u_{tip,x}$, where $u_{tip,y} > 0\, \text {mm}$ is satisfied, as the horizontal step length of the leg, denoted as $s_{x}$. In addition, the FEM-calculated stress distribution in Fig. 3d has demonstrated that, with the introduction of $k_{joint}$, the most deformations and stresses of the bent leg are evenly located in the flexible beams. This property could effectively improve the robustness and stability of the synthesized compliant leg.

###  Selection of the weighting factor $w_1$ and $w_2$

**Figure 4:**
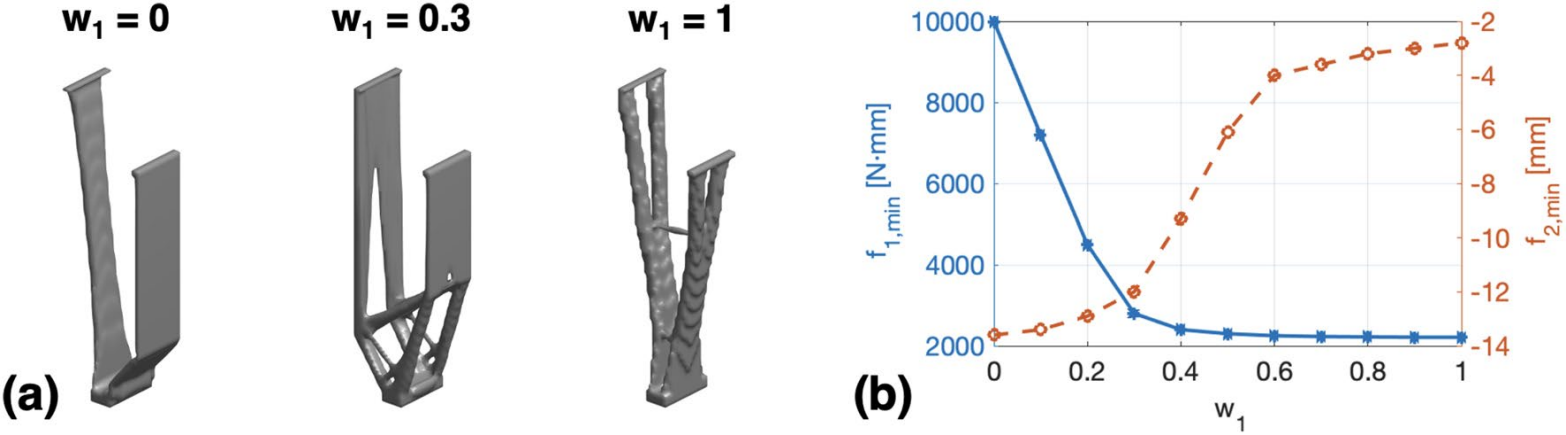
Compliant legs synthesized by using different weighting factors: (**a**) Synthesis results with $w_1=0,0.3,1$, (**b**) Relationship between the minimized objective functions ($f_{1,min}$ and $f_{2,min}$) and the weighting factor $w_1$.

To investigate the effect of different weighting factors on the optimized legs, we have tried 11 combinations of $w_1$ and $w_2$ ($w_1 = 0, 0.1, 0.2,..., 1$) in the design case of “Application to compliant leg design” section. The synthesis results are presented in Fig. 4, where $f_{1,min}$ and $f_{2,min}$ denote the minimized value of $f_{1}$ and $f_{2}$ in the optimization process, respectively. It can be seen that, when one of the weighting factors ($w_1$ or $w_2$) is too small, the created leg is either too flexible [$w_1=0$ in Fig. 4a] or too rigid [$w_1=1$ in Fig. 4a]. Herein, in order to find the most balanced weighting factor set, we have set the following selection criterion:

8 $$\begin{aligned} \left\{ \begin{aligned} f_{1,min}(w_1)< 1.5 \cdot f_{1,min}(w_1 = 1) \\ f_{2,min}(w_1) < 0.85 \cdot f_{2,min}(w_1 = 0) \\ \end{aligned} \right. \end{aligned}$$

From the $f_{1,min}$-$w_1$ curve and $f_{2,min}$-$w_1$ curve in Fig. 4b, it can be noticed that only the case of $w_1=0.3$ satisfies the criterion of (8). Therefore, we have chosen $w_1=0.3$ and $w_2=0.7$ in this work to perform the leg synthesis.

**Figure 5:**
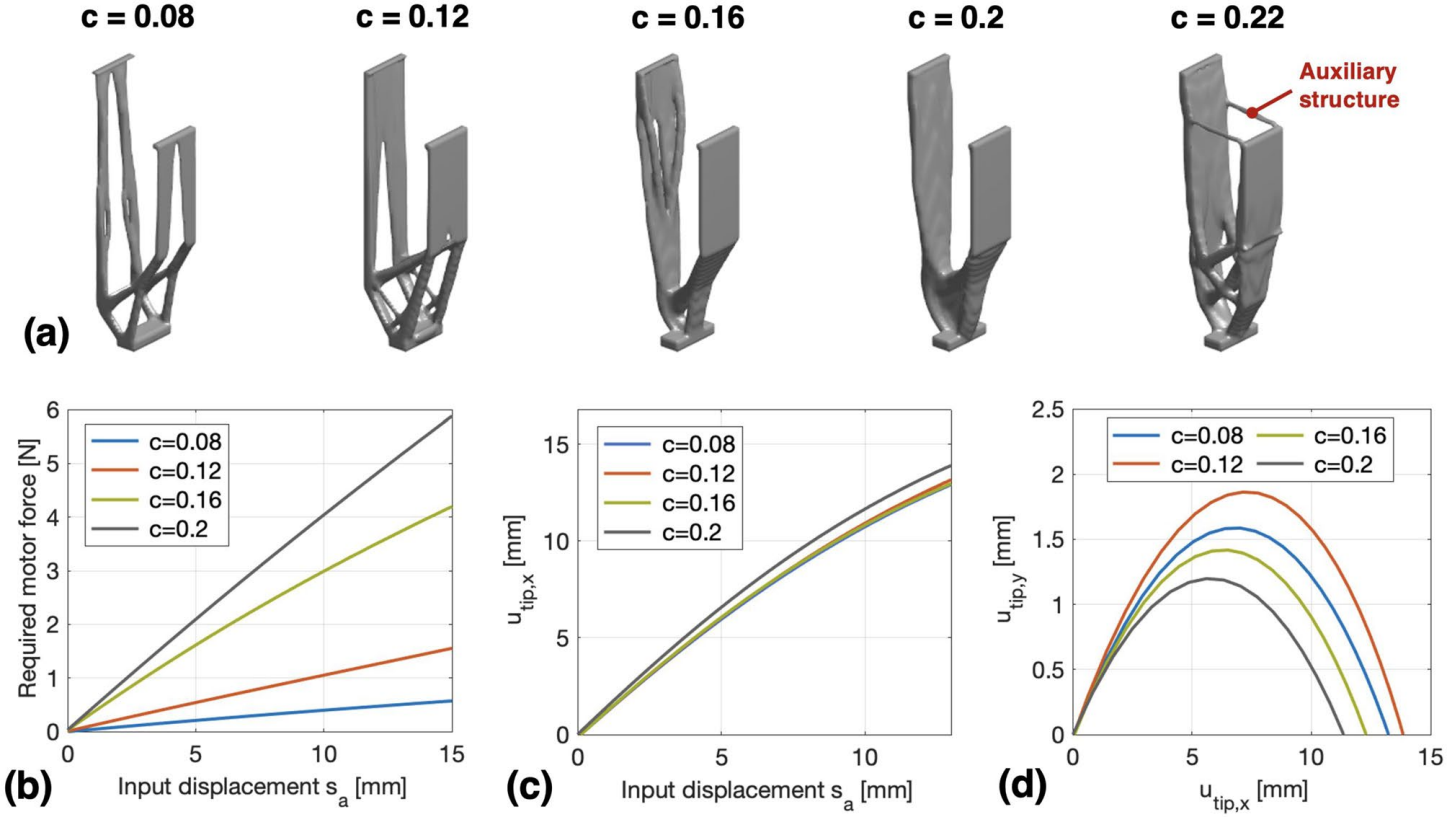
Compliant legs synthesized by using different volume constraints *c*: (**a**) The optimized leg shapes, (**b**) FEM-calculated actuation force (in N) for the realized legs, (**c**) FEM-calculated relationship between $s_a$ and the induced $u_{tip,x}$ for the realized legs, (**d**) FEM-calculated relationship between $u_{tip,x}$ and $u_{tip,y}$ during the two-stage bending motion of the legs.

### Effect of the volume constraint *c*

To analyze the function of the volume constraint *c*, we have performed the design case in “Application to compliant leg design” section with 5 different values of *c* (0.08, 0.12, 0.16, 0.2 and 0.22). The spring position $h_{joint}$ was set to 20 mm, while the other parameters used in the optimization process were the same as in Table 1.

The optimization results are reported in Fig. 5. It can be noticed that the realized leg structure generally becomes thicker as *c* increases, while the two flexible beams still remain for achieving bending motions. However, when the volume of the leg increases to a certain level (e.g., the case of $c = 0.22$), some auxiliary structures have inevitably emerged in the optimization process in order to meet the volume requirement. These auxiliary structures have connected the proximal ends of the two beams and greatly reduced the flexibility of the entire structure. From this point of view, the leg realized by $c = 0.22$ is considered as an inappropriate design and is not used for further mechanical analysis. Fig. 5b and c show the FEM-simulated actuation performance of the realized legs. We can see that, with the same input displacement $s_a$, the legs synthesized by different *c* produce similar tip motions $u_{tip,x}$ in x-axis, yet a higher *c* leads to the need for a greater actuation motor force. On the other hand, Fig. 5d shows that, the legs with different values of *c* can achieve different step lengths $s_x$ during the two-stage bending motion. Among them, the leg with $c = 0.12$ produces the largest step length ($s_x = 14\, \text {mm}$), while the leg with $c = 0.2$ has the smallest step length ($s_{x} = 11.5\, \text {mm}$).

### Effect of the spring position $h_{joint}$

To study the effect of the spring position $h_{joint}$ on the optimized legs, we have also performed the design case in “Application to compliant leg design” section with 5 different values of $h_{joint}$ (10 mm, 15 mm, 20 mm, 25 mm and 30 mm). The volume constraint *c* was set to 0.12, while the other parameters used in the optimization process were the same as in Table 1.

**Figure 6:**
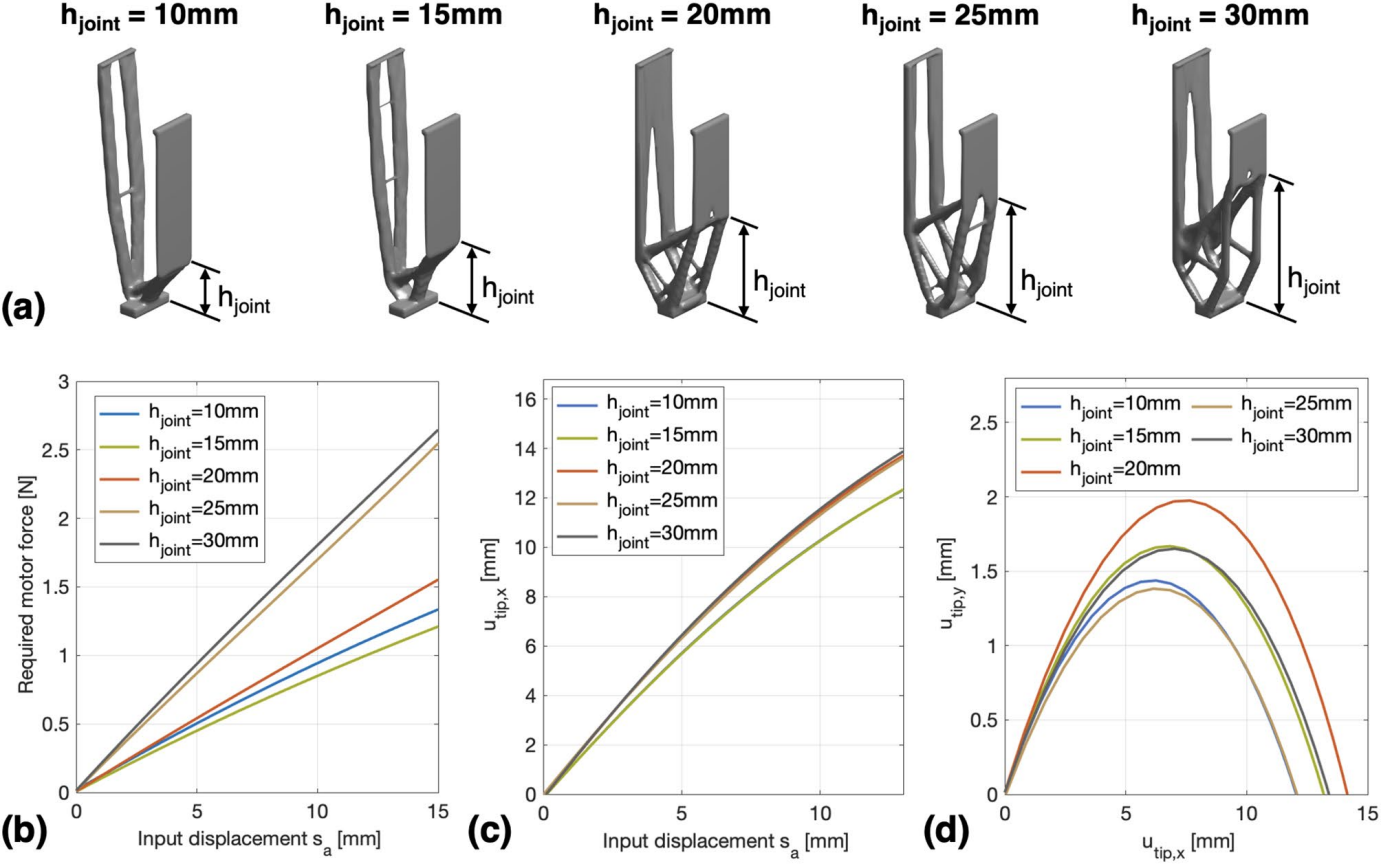
Compliant legs synthesized by using different spring positions $h_{joint}$: (**a**) The optimized leg shapes, (**b**) FEM-calculated actuation force (in N) for the realized legs, (**c**) FEM-calculated relationship between $s_a$ and the induced $u_{tip,x}$ for the realized legs, (**d**) FEM-calculated relationship between $u_{tip,x}$ and $u_{tip,y}$ during the two-stage bending motion of the legs.

Figure 6 shows the optimization results. It can be seen from Fig. 6a that, each optimized leg contains the combined structure of flexible beams and rigid foot as in the aforementioned design cases. However, the size of the synthesized rigid foot structure grows as $h_{joint}$ increases. This phenomenon can be explained by the spring $k_{joint}$, since its function is to introduce additional structural compliance (flexibility) in the optimized leg, and its application point can be interpreted as the compliant joint of the leg that connects the flexible and rigid parts^20^. Therefore, when the application point of $k_{joint}$ is shifted higher, the area of the rigid part will also be expanded in the optimization process. On the other hand, Fig. 6b and c show that, the same input displacement $s_a$ can generate similar $u_{tip,x}$ for different legs, while the legs optimized with $h_{joint} = 25\, \text {mm}$ and $h_{joint} = 30\, \text {mm}$ require greater actuation forces than the other legs. The main reason for that is the short length of the synthesized beams in these two legs, which reduces their flexibility in bending motions. In addition, as can be seen from Fig. 6d, the optimized legs with $h_{joint} = 10\, \text {mm}$ and $h_{joint} = 25\, \text {mm}$ produce the smallest step length ($s_x = 12.3\, \text {mm}$), while the largest step length ($s_x = 14\, \text {mm}$) is provided by the leg with $h_{joint} = 20\, \text {mm}$.

## Experiment

**Figure 7:**
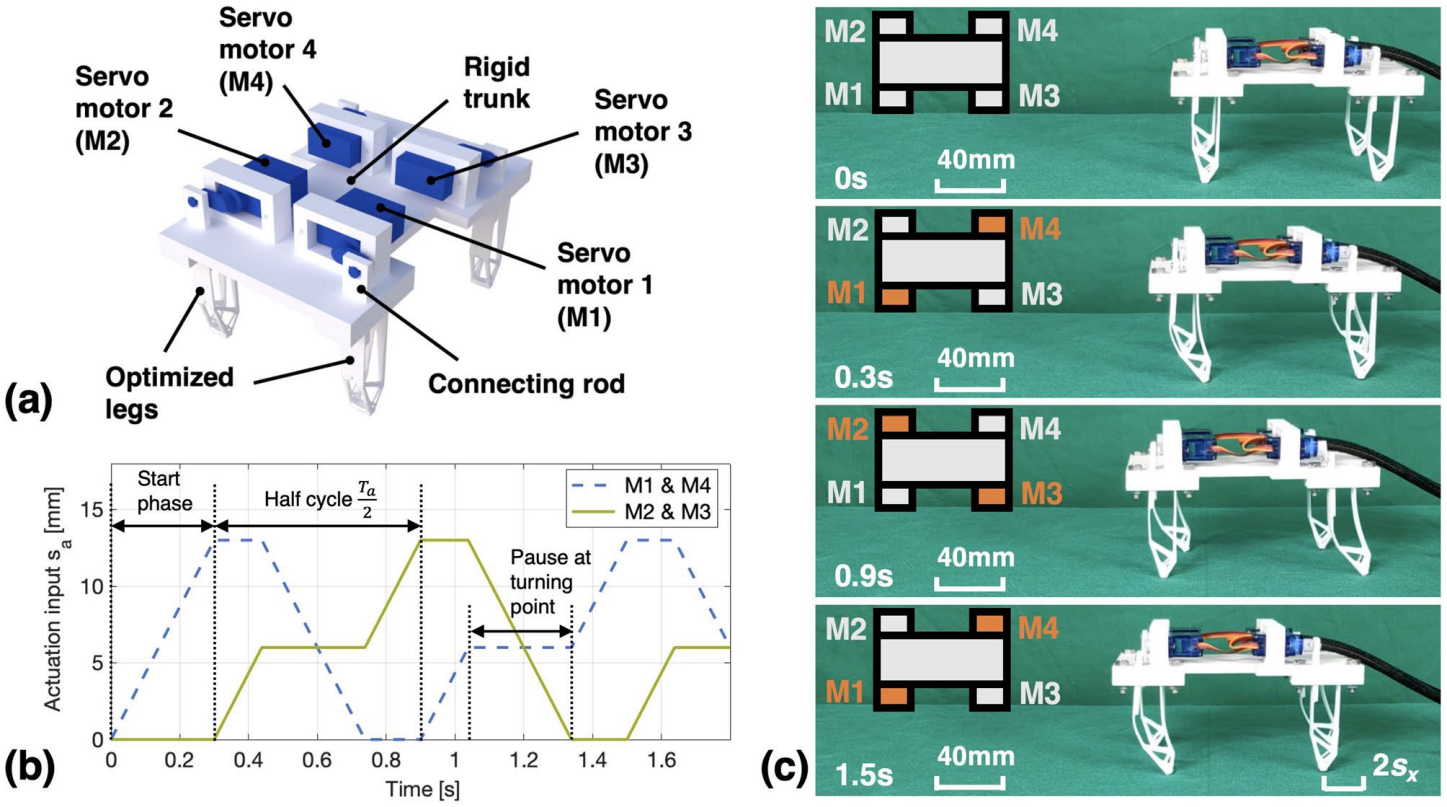
Experimental test of the walking performance of a 3D-printed quadruped robot with the optimized compliant legs: (**a**) Overview of the realized quadruped robot, (**b**) Actuation inputs for the two leg groups, (**c**) Generated walking motions of the robot. Here, the motor marked with orange color means that the connected leg is fully actuated ($s_a = 13\, \text {mm}$), while the gray color means that the connected leg is undeformed ($s_a = 0\, \text {mm}$).

In this section, in order to evaluate the feasibility of the proposed design method, we have created a 3D-printed quadruped robot with the optimized legs and tested its straight-line walking performance. Herein, the leg synthesized with $c = 0.12$ and $h_{joint} = 20\, \text {mm}$ (the case in “Application to compliant leg design” section) was used for experiment, since it produces the largest step length among all legs in this work [see Figs. 5d and 6d]. Fig. 7a provides an overview of the structure of the created quadruped robot, where the four compliant legs are mounted on a rigid trunk of the robot and actuated by four servo motors (SG90 Micro Servo), respectively. By introducing a horizontal sliding slot in the connecting rod, the vertical motion of the servo motor is used as the input displacement $s_a$ for the leg. In addition, a microcontroller (Arduino UNO) was also used in the experiment to control the actuation position of the servo motors.

To achieve continuous and stable straight-line walking of the robot, we first divided the robot’s legs into two motion groups according to its diagonal, i.e., the legs connected to the servo motors M1 and M4 were classified as one group, while the legs connected to the servo motors M2 and M3 belonged to the other group. The legs in the same group have the same motion pattern. Then, we used two series of actuation inputs [see Fig. 7b] to make the two leg groups perform periodic bending motions with a specific phase shift. It can be seen from Fig. 7b that, the cycle period of the two actuation inputs is $T_a = 1.2 \text {s}$, while the phase shift between them is a half cycle (0.6s). Here, the use of the first 0.3s is to initiate the walking mode of the robot by putting the two leg groups in fully bent ($s_a = 13\, \text {mm}$) and undeformed ($s_a = 0\, \text {mm}$) positions, respectively. After 0.3s, the input signals periodically switch the two leg groups back and forth between these two positions to realize forward walking motion of the robot. It should be mentioned that, a leg contributes to the forward motion of the robot only when it is actuated from the undeformed position to the fully bent position, while its 0.3s pause at the turning point of the bending motion [$s_a = 6\, \text {mm}$, also shown in Fig. 3c] allows the other fully bent legs to switch back to their undeformed position without pulling the robot backwards. Due to the long-strip contact surface between the foot tip and the ground, the robot can be supported by a pair of diagonal legs during the walking motion without tilting to the side. Since the distance the robot moves forward in a cycle is two step lengths ($2 \cdot s_x$), the theoretical walking speed $v_t$ can be calculated as:

9 $$\begin{aligned} v_t = \frac{2 \cdot s_x}{T_a} = \frac{2 \cdot 14 \text {mm}}{1.2 \text {s}} = 23.3 \text {mm/s} \end{aligned}$$

Figure 7c shows the walking gaits achieved by the 3D printed quadruped robot on a fabric-based surface. It can be seen that the robot is able to stand stably and move forward continuously with the provided actuation inputs. To quantitatively evaluate its walking performance, we have also measured the time required for the robot to walk 200 mm in the experiment. The test was repeated for five times and the measured average time is 9.2s. Hence, the measured walking speed $v_m$ of the robot can be obtained as:

10 $$\begin{aligned} v_m = \frac{200 \text {mm}}{9.2 \text {s}} = 21.7 \text {mm/s} \end{aligned}$$

which is $6.8\%$ slower than $v_t$. Here, the difference between the calculated and measured walking speed is mainly caused by the slight slippage between the 3D-printed legs and the fabric when the robot moves. In addition, we have also conducted walking tests with payload and the results show that the robot can stably move forward with a maximum weight of 800g (8 times of its self-weight). With these experimental results, the feasibility of the proposed method for designing compliant robotic legs is successfully verified.

## Discussion

In this article, we have presented a novel method for designing fully compliant legs for quadruped robots, which is based on the topology optimization approach. Experimental evaluation was conducted and verified the feasibility of the proposed method. Compared to the rigid-link-based robotic legs^2,3^, the monolithic compliant legs realized in this work have greatly simplified the leg assembly process and also introduced additional structural compliance to the quadruped robotic system. On the other hand, thanks to the topology optimization algorithm, the proposed method is able to achieve the automatic design of the holistic leg structure, and the generated solution has a biomimetic continuum shape. In addition, different from other topology optimized flexible bending structures^27–29^, the presented work has employed a multi-objective algorithm that can successfully achieve a balance between the structural stiffness and bending flexibility in the compliant leg structure.

Nevertheless, this work can still be improved in several aspects. For instance, to achieve faster walking speed and improve the ability to traverse obstacles in the complex environment, we will utilize a larger design domain in our future work to perform leg synthesis. In this way, the created robot leg will be optimized with a larger step length in both horizontal and vertical direction. In addition, more advanced algorithms, such as genetic algorithms^30–32^, can be introduced to improve the performance of the proposed multi-objective process. Furthermore, we also plan to incorporate a geometrically nonlinear model into the proposed optimization algorithm to achieve large-displacement synthesis of compliant legs with specific motion curves.

## Supplementary Information

Supplementary Video 1.

## Data Availability

The MATLAB codes and the datasets generated or analyzed during this study are not publicly available due to the confidentiality agreement of the Institute of Micro Technology and Medical Device Technology, but are available from the corresponding author on reasonable request.
